# From Waste to Resource: Biosolids from Sludge Treatment Wetlands as Biofertilizers and Biostimulants

**DOI:** 10.1021/acsenvironau.6c00070

**Published:** 2026-04-21

**Authors:** Ana Cano-Larrotta, Luisa Massaccesi, Enrica Uggetti, Mirko Cucina

**Affiliations:** † Department of Civil and Environmental Engineering (DECA), Group of Environmental Engineering and Microbiology (GEMMA), Barcelona School of Civil Engineering (ETSECCPB), 16767 Universitat Politècnica de Catalunya-BarcelonaTech (UPC), Campus Nord, C. Jordi Girona 1-3, Building D1, E-08034 Barcelona, Spain; ‡ National Research Council of Italy, 9304 Institute for Agricultural and Forest Systems in the Mediterranean, Via della Madonna Alta 128, Perugia 06128, Italy

**Keywords:** biosolids, biostimulant, nutrient leaching, soil fertility, sludge treatment wetlands, waste management

## Abstract

This research assessed biosolids from sludge treatment wetlands (STW) as possible biofertilizers, comparing them with digestate, compost, and microalgae biomass in greenhouse trials involving three crops (i.e., lettuce, radish, and ryegrass). Additionally, the potential biostimulant effects were evaluated through bioassays conducted under controlled conditions using water extracts of the biosolids. The biosolids showed a notable increase in radish growth compared with the unfertilized control, with increases of 93% and 95% in fresh and dry weight, respectively. The protein content of all crops grown under biosolids treatment was similar to that of microalgae, and it exceeded the control treatments by 6%, 9%, and 17% for lettuce, ryegrass, and radish, respectively. Furthermore, using biosolids reduced nutrient loss through leaching (i.e., N mineral forms), which was observed in urea treatments. Phytohormone-like bioassays revealed that biosolids extracts showed gibberellin-like activity, aiding seed germination and growth. Biosolids extracts also delayed senescence by slowing chlorophyll breakdown in wheat leaves and stimulated secondary root formation in bean seedlings. Overall, the results demonstrated the dual role of STWs’ biosolids as biofertilizers and biostimulants, with performance comparable to urea and most of the biofertilizers used for comparison. Future studies should include field trials to confirm agronomic performance under real conditions.

## Graphical abstract

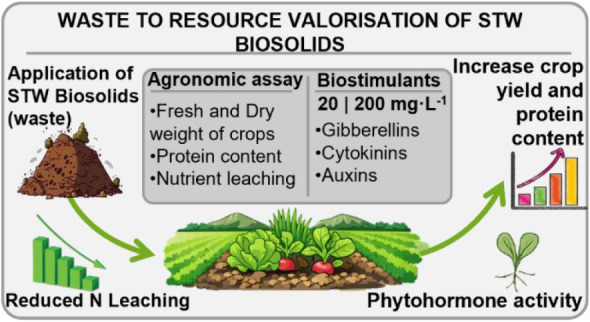

## 1 Introduction

In response to increasing food demand, conventional farming methods have become increasingly reliant on chemical fertilizers. Extensive use of chemical fertilizers potentially harms ecosystems, i.e., through nutrients leaching into groundwater, eutrophication of surface waters, significant greenhouse gas emissions, and adverse effects on human health.^1,2^ Additionally, the rising costs of producing chemical fertilizers due to energy-intensive production processes and limited resources such as natural gas and phosphate rock highlight the urgent need for sustainable alternatives.^3–5^

Recycled biofertilizers, including compost, digestate, and microalgae derived from wastewater treatment, have been studied as substitutes for, or supplements to, inorganic fertilizers.^6–8^ By promoting material reuse and recycling, reducing waste generation, and minimizing raw material extraction, the use of recycled biofertilizers in agriculture represents a strategy closely aligned with the Sustainable Development Goals (SDGs) 2 (Zero Hunger), 14 (Life Below Water), and 15 (Life on Land), among others.^5^ In addition, biofertilizers are widely recognized for their lower environmental impact, i.e., reduced nutrient leaching into groundwater and runoff into surface water that can cause eutrophication. For instance, organic nitrogen, predominant in compost, in the solid fraction of digestate, and in microalgae biomass, reduces the risk of nitrogen leaching into groundwater compared with synthetic fertilizers such as urea.^9^

While biofertilizers can supply essential nutrients and enhance soil organic matter,^10,11^ their effectiveness varies depending on feedstock composition, processing methods, and nutrient availability.^12^ Among the possible biofertilizer sources, biosolids from sludge treatment wetlands (STWs) are a promising alternative. Unlike traditional sewage sludge treatment, STWs use natural processes to stabilize sludge, producing a low-cost bioproduct that is highly sanitized and enriched with stabilized organic matter and nutrients. However, its agricultural reuse requires proper characterization, risk assessment, and compliance with current regulations.^10^ The low levels of heavy metals and pathogenic microorganisms in biosolids derived from STWs make them a competitive and sustainable alternative to conventional biosolids produced by other sludge treatment processes.^13^ However, despite recent studies suggesting that STW-derived biosolids often exhibit advantageous properties,^10,11,13^ this product remains underexplored, and this research gap hinders its wider adoption.

In addition to serving as a rich source of nutrients for soil fertility, biosolids have demonstrated biostimulant effects due to the presence of humic substances and hormone-like compounds, such as cytokinins and auxins, which can promote plant growth and delay senescence.^14^ Nevertheless, the biofertilization and biostimulant potential of biosolids may be constrained by several factors related to their origin and treatment. If biosolids are insufficiently stabilized or managed, they may contain residual pathogens, excess nutrients, heavy metals, and organic contaminants, potentially leading to environmental impacts such as nutrient leaching or soil accumulation. There has also been increasing attention to emerging contaminants, including pharmaceuticals, personal care products, and per- and polyfluoroalkyl substances (PFAS), which may persist through wastewater and sludge treatment processes and raise concerns about long-term soil–plant interactions.^15–18^ Furthermore, variability in sludge composition and treatment efficiency can influence nutrient availability and biological activity, contributing to heterogeneous agronomic responses. In this context, the number of studies directly assessing the biostimulant properties of biosolids from STW systems remains very limited. This highlights the need for a comprehensive investigation into the agronomic potential of STW-derived biosolids.

Therefore, the objective of this study was to evaluate the fertilizing and biostimulant properties of STW-biosolids under controlled conditions and to compare them with other biofertilizers (i.e., digestate, compost, and microalgae biomass). Lettuce, radish and ryegrass were tested to evaluate the fertilizing value. In addition, nutrient leaching (i.e., mineral N) from soil following biofertilizer application was evaluated. Biostimulant properties were evaluated in biosolids water extracts employing assays to assess auxin-, gibberellin-, and cytokinin-like activities. Consequently, this study hypothesized that STW biosolids can serve a dual function: (i) acting as a nutrient-rich biofertilizer capable of supporting the growth of three different crops and reducing the environmental risks related to soil fertilization, and (ii) functioning as a natural biostimulant source with activities similar to those of auxins, cytokinins, and gibberellins.

## 2 Materials and Methods

### 2.1 Soil and Biofertilizers

#### 2.1.1 Soil Preparation

For the experiments, soil was collected from an agricultural field near Perugia, Central Italy (43° 5′ 6″ N–12° 24′ 9″ E), cultivated with olive trees. The field is approximately 400 m above sea level (m.a.s.l.), with an average temperature of 24 °C and an average annual precipitation of 805 mm. The soil has never received pesticide or fertilizer treatments. Prior to sampling, several auger holes were drilled across the field to assess the homogeneity of the pedological conditions. Soil was collected from the cultivated Ap horizon, comprising the Ap1 and Ap2 subhorizons within the same depth interval (0–25 cm), using a manual soil shovel. Ten soil subsamples, each approximately 10 kg, were collected across the field after removing surface vegetation and plant residues. These subsamples were then combined to produce a representative, homogeneous bulk soil sample of approximately 100 kg, a quantity needed for the pot experiments. Before the fertilization tests, the soil was air-dried, and a subsample (1 kg) was characterized after sieving it at 2 mm (Table S1, Supporting Information). According to the World Reference Base for Soil Resources (WRB), the soil was classified as a Cambisol. The textural class determined from a particle-size analysis was clayey-loam, consisting of 6% coarse sand, 26% fine sand, 38% silt, and 30% clay. Its characteristics included a pH of 7.8, an electrical conductivity (EC) of 0.23 mS·cm^–1^, a total organic carbon (TOC) content of 1.2%, a total nitrogen (N) content of 0.19%, and a water-holding capacity (WHC) of 36.4% (Table S1). The air-dried soil was manually crushed for the fertilization trials, and stones and debris were removed until a homogeneous consistency was achieved. Before starting the experiments, the soil was moistened with deionized water to achieve 60% WHC, a moisture level commonly used in controlled soil/pot studies to ensure adequate water availability without saturations.^19^

#### 2.1.2 Biofertilizer Preparation and Characterization

The biofertilizers (BFs) used in this study included biosolids, microalgae biomass, compost, and the solid fraction of digestate. The biosolids were sourced from sludge treatment wetlands (STW), and they underwent a final stabilization period of 6 months. The treatment system, the origin, and the quality of biosolids are described in detail in Cano-Larrotta et al.^10^ The microalgae biomass was collected from a wastewater treatment pilot plant.^20^ The compost was produced from olive pruning and olive mill waste through a thermophilic composting process. The solid fraction of the digestate was obtained from the solid/liquid separation of digestate derived from a mesophilic anaerobic digestion facility processing livestock manure. All BFs were freeze-dried and sieved at 0.5 mm prior to experiments and for chemical characterization.

The BFs were characterized in terms of total solids (%TS), volatile solids (%VS/%TS), pH (measured using a Basi 20 + pH-Meter, Crison Instruments, Barcelona, Spain), and electrical conductivity (EC) (measured using a Basi 30 + EC-Meter, Crison Instruments, Barcelona, Spain). Total solids were determined by drying a few grams of fresh sample in an oven at 105 °C until a constant mass was achieved. Subsequently, the dried samples were incinerated at 550 °C for 20 min to determine organic matter content, which was expressed as volatile solids (%VS/%TS).^10^ pH and EC were determined on aqueous extracts prepared from fresh samples of biofertilizers (2:20 w/v ratio). TOC and total N were analyzed using an elemental analyzer (MacroCUBE CNHS, Elementar Italia, Lomazzo, Italy). C/N ratio was then calculated. Dissolved organic carbon (DOC) was quantified from aqueous extracts following the methodology of Cano-Larrotta et al.^11^ The extracts were prepared by weighing 2 g of freeze-dried sample and dissolving it in 10 mL of deionized water. The solution was stirred for 24 h, centrifuged at 5,000 rpm for 10 min and filtered through a 0.45 μm filter. DOC was then quantified by using the dichromate oxidation method.

### 2.2 Fertilization Assay

The fertilization trials were conducted in the greenhouse of the Institute for Agriculture and Forestry Systems in the Mediterranean, located in Perugia, central Italy (43°05′36.3″ N 12°21′49.3″ E), using three different crops: lettuce (*Lactuca sativa*, L. var. Romana), ryegrass (*Lolium hybridum* Lam.), and radish (*Raphanus sativus* L. var. Cherry Belle). These crops were selected for their diverse characteristics. Lettuce and radish are commonly used in fourth-range production, which is a suitable market for biofertilizers given the sector’s high economic value. Additionally, lettuce and ryegrass enabled evaluation of fertilizing effects on aerial (leaf-producing) crops, while radish provided insights into fertilizing potential for hypogeal organs (roots). Finally, ryegrass was included to assess the fertilizing potential of biofertilizers in monocotyledons, rather than in lettuce and radish, which are dicotyledons.

To determine whether biosolids from STW have comparable or superior biofertilizing capacity to other organic materials used as N biofertilizers, six different treatments were evaluated:

i CTR – Unfertilized negative control (−);
ii UR – Urea, positive control (+);
iii BSD – Biosolids;
iv AL – Microalgae biomass;
v CMP – Compost;
vi DIG – Digestate solid fraction.

Fertilization rates for all crops were calculated based on the nitrogen nutritional requirement of each crop, which was found to be 200 kg N·ha^–1^,^21,22^ the nitrogen content of each biofertilizer (Table 1), soil density (1.3 kg·dm^–3^), and the dimensions of the pots. Pot dimensions were selected based on crop-specific growth habits, planting method (transplanting vs direct sowing), and expected root system development to minimize root restriction during the experimental period. Therefore, the nitrogen requirements for the crops were determined to be 0.067 gN pot^–1^, 0.102 gN pot^–1^, and 0.113 gN pot^–1^ for radish, ryegrass, and lettuce, respectively (Figure 1). Phosphorus and potassium fertilization were not provided, as soil analyses indicate high availability of both elements (Table S1). Therefore, only nitrogen was considered in the fertilization assay.

**Table 1:** Fertilizer Application Rates per Pot Based on Nitrogen Requirements Using Different Organic Materials^a^

		Mass of fertilizer per pot				
Nitrogen requirement	Crops	UR (+)	BSD	AL	CMP	DIG
200 kg N·ha^–1^	Lettuce	0.25	3.48	1.51	2.14	5.30
	Ryegrass	0.22	3.16	1.37	1.94	4.81
	Radish	0.14	2.06	0.89	1.26	3.13

a UR (+) as the positive control using inorganic fertilizer (urea), BSD: biosolids, AL: microalgae, CMP: compost, and DIG: digestate.

**Figure 1:**
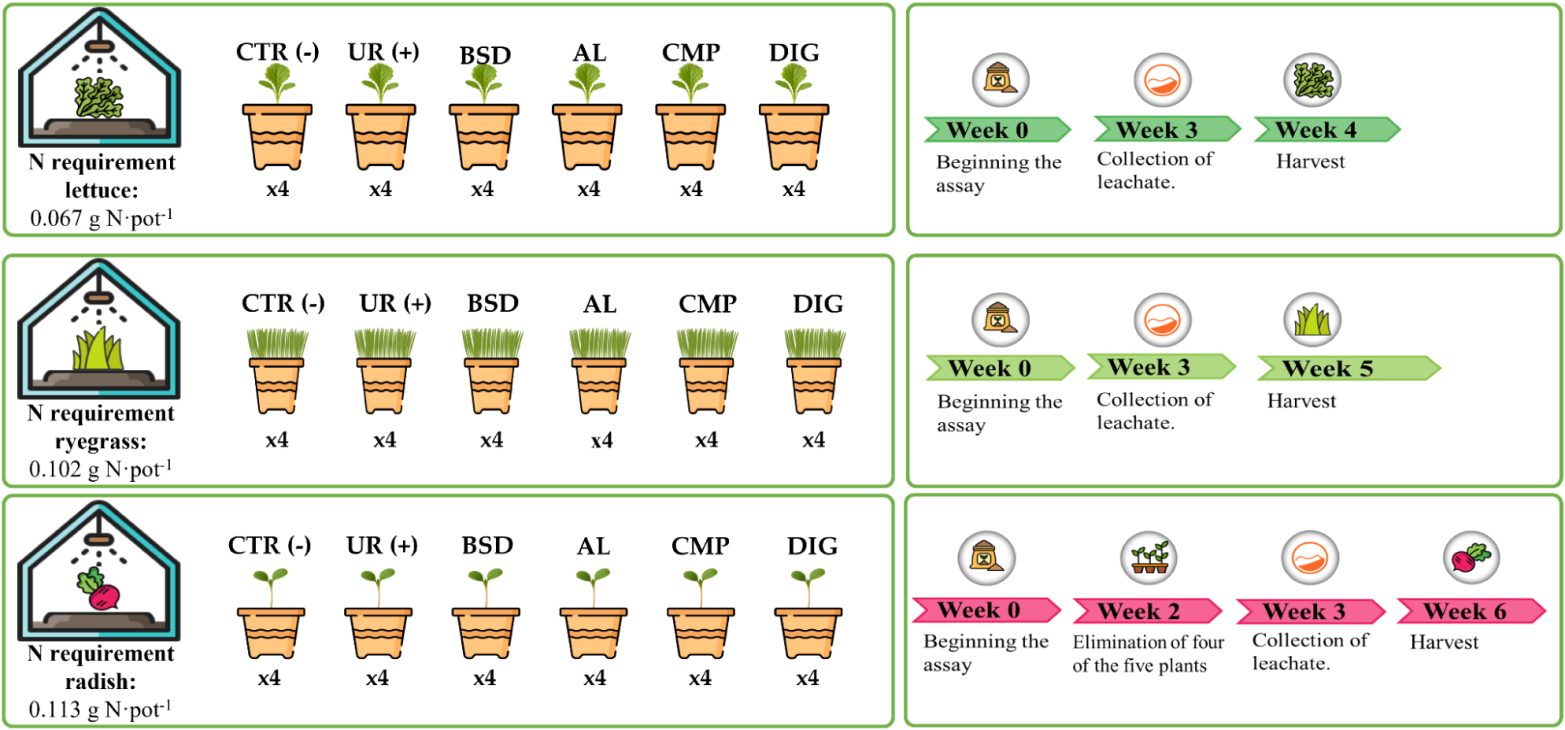
Graphic outline and timeline for setting up agronomic trials for three crops: lettuce, ryegrass, and radish. Each crop received six treatments: CTR (−) as the negative control without fertilizer; UR (+) as the positive control using inorganic fertilizer (urea); BSD biosolids; AL microalgae; CMP compost; and DIG digestate.

For lettuce, 1.10 kg of soil was placed in each plastic pot (13 cm high × 8 cm in diameter). The respective amounts of each BFs (UR, BSD, AL, CMP, and DIG) were weighed on a dry matter basis as specified in Table 1, mixed thoroughly into the soil, and lettuce seedlings were transplanted into each treatment pot. For ryegrass, 1 kg of soil was placed in each plastic pot (12 cm high × 9 cm in diameter), and the BFs were incorporated in the same manner as for lettuce (Table 1). Subsequently, the ryegrass sowing density was calculated by scaling the recommended field seeding rate (110 kg·ha^–1^) to the pot surface area, resulting in a seed quantity of 60 mg of seeds per pot.^23^ This quantity was applied to each pot, then covered with soil. Finally, for radish, 0.65 kg of soil was placed in each plastic pot (8 cm high × 8 cm in diameter). The corresponding BFs were homogenized with the soil (Table 1), and five radish seeds were placed in each pot for the treatments and covered with soil (2 cm deep). After germination (2 weeks), the plants in each treatment were thinned, leaving only one plant with well-developed foliage, as suggested by Lima et al.^24^ For all crop studies, each treatment was replicated four times (Figure 1).

Throughout the experiment, all pots were irrigated manually daily. During the first 2 weeks, 30 mL of water per pot was applied to support seed germination and early establishment. Thereafter, 50 mL per pot was supplied daily. Irrigation volume was adjusted based on visual plant water status (e.g., the onset of leaf wilting), while maintaining the same regime across treatments to ensure adequate moisture and minimize nutrient losses through leaching.

### 2.3 Harvest and Processing

Lettuce, ryegrass, and radish crops were harvested weeks 4, 5 and 6, respectively (Figure 1), and the fresh weight of all crops was recorded. Lettuce was harvested by cutting the entire plant at the basal stem (plant collar/crown), ryegrass was harvested by simulating a complete cut of the aerial biomass at collar height, and radish was harvested by separating the root system from the shoot tissues, which were discarded. Subsequently, the samples were dried in an oven at 60 °C for 48 h, then weighed to determine their dry weight.

Protein content of lettuce, ryegrass, and radish was determined from dried samples by measuring total N with an elemental analyzer (MacroCUBE CNHS, Elementar Italia, Lomazzo, Italy) and multiplying the result by 6.25, a standard conversion factor for protein.^25^

### 2.4 Leachate Test

Nitrogen leaching caused by overfertilization significantly contributes to pollution in both surface and groundwater.^6^ To assess the extent of nitrogen leaching from each treatment in this study, leachate samples were analyzed for nitrite (NO_2_^–^), nitrate (NO_3_^–^), and ammonium (NH_4_^+^). Leachates from all the pots were obtained after 3 weeks from the beginning of the fertilization trials by adding deionized water to allow leaching from the bottom of the plastic pots (100 mL of water for each pot) as suggested by Cucina et al.^26^ The analysis of NO_2_^–^ and NO_3_^–^ was conducted according to Umeda et al.^27^ For NO_3_^–^, all samples were diluted at a ratio of 1:10 with deionized water. A 2 mL volume of the diluted sample was taken, to which 0.2 μL of Griess reagent and two metallic Zn beads (1 g) were added. The mixture was then shaken and left to react for 30 min. Afterward, the absorbance was measured at a wavelength of 540 nm (UV1900i Spectrophotometer, Shimadzu Italia, Milano, Italy). For NO_2_^–^ analysis, the same procedure was followed, but without diluting samples and without adding metallic Zn beads. Concentrations of NO_2_^–^ and NO_3_^–^ were expressed as mg·L^–1^ based on calibration curves prepared with KNO_2_ and KNO_3_, respectively. Ammonium-N was measured following the modified methodology of Tartari and Mosello (1997). A volume of 2.5 mL of the sample was taken, followed by the addition of 0.1 mL of solution II (sodium nitroprusside dihydrate) and 0.1 mL of solution IV (tribasic sodium citrate dihydrate with sodium hydroxide/sodium dichloroisocyanurate). The mixture was shaken, and the absorbance was subsequently measured at a wavelength of 680 nm (UV1900i Spectrophotometer, Shimadzu Italia, Milano, Italy). Ammonium-N concentration was expressed as mg·L^–1^ based on an ammonium-N calibration curve prepared with NH_4_Cl.

### 2.5 Biostimulant Tests

Bioassays were performed to assess the plant growth-promoting properties of extracts from BSD, AL, CMP, and DIG. These bioassays were designed to determine whether substrates contain molecules with activity similar to auxins, cytokinins, and gibberellins, i.e., phytohormones vital for plant growth and development.^29^ For the biostimulant bioassays, water extracts of all substrates were used (1:10 w/v in deionized water, filtered at 0.2 μm to avoid any biostimulant effect resulting from the presence of microorganisms), which were tested at two concentrations based on the DOC concentration (Table 2): 20 mg·L^–1^ and 200 mg·L^–1^ of DOC. These concentrations were selected based on the study by Scaglia et al.,^30^ which reported biostimulant activity of vermicompost across a similar range of DOC concentrations.

**Table 2:** Physicochemical Properties of Biofertilizers[Table-fn tbl2fn1] [Table-fn tbl2fn2]

Parameters	Units	Biosolids		Microalgae		Compost		Digestate	
Total solids	%	50 ± 0.9	ab	12 ± 0	c	89 ± 1	a	13 ± 1	bc
Volatile solids	%VS/%TS	47 ± 1	b	81 ± 0	a	85 ± 1	a	83 ± 1	a
pH	-	5.9 ± 0.1	ab	6.7 ± 0	ab	5.8 ± 0.1	b	9.4 ± 0	a
Electrical conductivity	dS m^–1^	1.6 ± 0	b	1.1 ± 0	c	8.6 ± 0.1	a	1.6 ± 0	b
Total organic C	%	23 ± 0	b	45 ± 0	a	46 ± 0	a	41 ± 0	ab
Total N	%	3.2 ± 0	c	7.5 ± 0	a	5.3 ± 0.1	b	2.1 ± 0.1	d
C/N	-	7.04 ± 0.08	c	6 ± 0.02	d	8.7 ± 0.09	b	14.9 ± 1.4	a
DOC	g·kg^–1^	0.8 ± 0.1	b	14 ± 1.8	a	1.8 ± 0.1	b	15 ± 1.4	a

i Mean value ± SD, n = 3. Different letters indicate significant differences. DOC: dissolved organic C.

ii Data are expressed in dry weight, except for total solids, electrical conductivity and pH, which were determined from fresh samples, and DOC, which was quantified from aqueous extracts prepared from freeze-dried material.

The tests included: (i) a germination index (GI) test to evaluate gibberellin-like activity in cress seeds (*Lepidium sativum*); (ii) the degradation of chlorophyll in detached wheat (*Triticum aestivum* *L.*) leaf tissues to evaluate cytokinin-like activity; and (iii) the formation of secondary roots in mung beans (*Vigna radiata* *L*.) to assess auxin-like activity. All these tests followed the methodology established by Ruales et al.^29^

#### 2.5.1 Germination Index of Cress Seeds

Gibberellins are a class of plant hormones that play a crucial role in various developmental processes, including seed germination, dormancy, flowering, leaf expansion, stem elongation, fruiting, and leaf senescence.^31^ To assess the effectiveness of biostimulant extracts in promoting seed germination, the germination index (GI) quantifies their efficacy while accounting for phytotoxic effects of the substrate.^32^ Extracts of BSD, AL, CMP, and DIG were tested on cress seeds (*Lepidium sativum*) to evaluate the germination response to the two selected concentrations of the extracts (20 mg·L^–1^ DOC and 200 mg·L^–1^ DOC). For each treatment and concentration, including a negative control (deionized water), five 90 mm Petri dishes lined with Whatman No. 5 filter paper were employed, with 10 cress seeds placed in each dish. One mL of the treatment extract at each concentration was applied to the filter paper; the dish was sealed and incubated in the dark for 48 h. Finally, the GI for each treatment was determined using the following equation^29^ (eq 1):

1 $$\mathrm{GI}\left(\%\right)=\frac{G\times L}{G_{\left(C^{-}\right)}\times L_{\left(C^{-}\right)}}\times 100$$

where G and L represent the number of germinated seeds and the radicle length, respectively, and $G_{\left(C^{-}\right)}$ and $L_{\left(C^{-}\right)}$ denote the corresponding parameters for the negative control.

#### 2.5.2 Chlorophyll Retention Assay on Wheat Leaves

Cytokinins are a class of essential plant hormones involved in all aspects of plant growth and development. Specifically, they are vital for cell division and delaying senescence, mainly by preventing chlorophyll degradation in detached leaf tissues.^33^ To assess the cytokinin-like effect, the amount of chlorophyll retained in wheat leaves (*Triticum aestivum* L.) was measured when substrate extracts at concentrations of 20 mg·L^–1^ DOC and 200 mg·L^–1^ DOC were applied. This approach may provide a reliable means of determining whether the cytokinins present in substrates effectively delay senescence. The wheat plants used to obtain leaf tissue for this experiment were harvested during flowering (third week of May 2024) in a field located near Perugia (43° 5′ 8″ N–12° 24′ 8″ E, Umbria, Central Italy) managed according to organic practices. Leaves were collected 35 mm below the apical tip, following established protocols to ensure uniform physiological tissue age,^29^ and cut into 10 mm segments. Five leaf segments were placed into glass vials (2 cm diameter × 5 cm height), each containing 5 mL of the corresponding extract, including a negative control (deionized water), and sealed with Parafilm. Five independent vials (replicates) were prepared for each treatment and concentration. The vials were incubated in a dark growth chamber at 20 °C for 4 days. After incubation, the leaves were removed from the vials, dried with absorbent paper, and transferred to centrifuge tubes containing 8 mL of 80% ethanol. All tubes were placed in a water bath at 80 °C for 10 min. Following cooling to room temperature, the samples were diluted with 80% ethanol to a total volume of 10 mL. The chlorophyll extract was transferred into a spectrophotometric cuvette, and any leaf residue was discarded. The absorbance was measured at a wavelength of 645 nm (UV1900i Spectrophotometer, Shimadzu Italia, Milano, Italy). At the end of incubation, chlorophyll degradation was compared with a negative control (deionized water) and a positive control prepared with the commercial Zeatin cytokinin (Merck KGaA, Darmstadt, Germany) at 1 mg·L^–1^. Results were expressed as % of the negative control.

#### 2.5.3 Adventitious Root Induction in Mung Beans

Auxins are vital phytohormones essential for plant development and growth, playing a key role in cell cycle progression and breaking bud dormancy. Furthermore, this phytohormone promotes shoot growth, root meristem development, and the positioning of lateral organ primordia.^33^ Auxin-like activity in substrate extracts was assessed by evaluating their capacity to induce rooting in mung bean (*Vigna radiata* L.) stem segments obtained from germinated seedlings. Bean seeds were sown in plastic trays filled with sterile, moistened perlite and placed in a greenhouse for 15 days until germination. After this incubation period, five homogeneous bean seedlings were selected and cut to obtain stem segments, which were then used as cuttings for each treatment and extract concentration (20 mg·L^–1^ DOC and 200 mg·L^–1^ DOC). Each seedling was placed in a glass vial (2 cm diameter and 8 cm height). Then, 5 mL of each extract at its respective concentration was added to each vial. Each seedling was incubated in a culture chamber at a constant temperature of 25 °C under a continuous controlled photoperiod (16 h light, 8 h dark) for 7 days. At the end of incubation, the number and weight of roots produced at each treatment and concentration was compared with a negative control (deionized water) and a positive control prepared by using a commercial auxin, indole-3-butyric acid (IBA) (Merck KGaA, Darmstadt, Germany), at a concentration of 1 mg·L^–1^. Results were expressed as % of the negative control.

### 2.6 Statistical Analysis

The experimental results were analyzed statistically using Minitab software, version 22.0.0. The results were presented as the mean ± standard deviation. The normality and homoscedasticity of the data were assessed using the Anderson-Darling test. After confirming the normality of the data, one-way analysis of variance (ANOVA) with Tukey’s post hoc tests was used to evaluate differences among treatments in agronomic trials, including protein content, leachate analyses, and biostimulant assays. The graphs display the mean values, with error bars representing the standard deviation. Each treatment was compared to its respective control.

## 3 Results and Discussion

### 3.1 Biofertilizers Characterization

Table 2 shows the physicochemical characteristics of the BFs studied. Biosolids and compost had high %TS contents (i.e., 50% and 89%, respectively), compared to microalgae and digestate. The biosolids exhibited a significant total N (TN) content of 3.2%, consistent with findings by Cano-Larrotta et al.^10^ suggesting that biosolids serve as an alternative N source in agriculture.^34^ The other BFs showed TN levels ranging from 2.1% (digestate) to 7.5% (microalgae), in line with the relevant literature dealing with on BFs.^35^

pH and EC values of the biofertilizers differed significantly among treatments. Biosolids derived from STW had a slightly acidic pH of 5.9, whereas compost and microalgae had nearly neutral pH values of 5.8 and 6.7, respectively. In contrast, the digestate was alkaline, with a pH of 9.4, consistent with the literature.^10,11^ The EC values were moderate in the biosolids, microalgae, and digestate, measuring 1.6 dS·m^–1^, 1.1 dS·m^–1^, and 1.6 dS·m^–1^, respectively, whereas compost exhibited significantly higher values at 8.6 dS·m^–1^. These moderate EC values are generally considered safe for crops, whereas elevated EC levels in compost may increase salinity risk, particularly for sensitive crops such as lettuce,.^26–28^

The C/N ratio determines whether biofertilizers act as a nitrogen source or sink during decomposition. In this study, biosolids (7.04), microalgae (6), and compost (8.7) exhibited relatively low C/N ratios, which promote rapid nitrogen mineralization and an immediate supply of available nitrogen for plants. Conversely, the digestate had a higher C/N ratio of 14.9, which could prevent nutrient release and potentially promote temporary nitrogen immobilization by soil microorganisms.^36^

Compost, microalgae, and digestate exhibited high total organic C contents of 46%, 45%, and 41%, respectively, while biosolids contained a lower TOC content (i.e., 23%). TOC contents were also reflected in VS values, which were significantly higher in compost, microalgae, and digestate compared to biosolids. This difference in carbon content among the BFs may be due to processes like oxidation of organic matter during wastewater treatment, composting, and anaerobic digestion, among others. Overall, stabilizing biosolids in STW might have lowered their carbon content.^37^ Finally, the DOC content for the BFs was 15 g·kg^–1^ for digestate, 14 g·kg^–1^ for microalgae, 1.8 g·kg^–1^ for compost, and 0.8 g·kg^–1^ for biosolids (Table 2). The lower DOC content in biosolids compared to other BFs could result from the maturation of organic matter in STWs.^10^ Conversely, digestate and microalgae contained a significant proportion of labile organic matter, likely due to the absence of an aerobic maturation of the BFs.^5^

Notably, biosolids met the legal requirements established by the European Fertilisers Regulation 1009/2019 for solid organic fertilizers (Product Function Category 1­(A)­I), which are set to the minimum values of 15% and 2.5% d.w. for TOC and TN, respectively, as per Annex I of Regulation 1009/2019.

### 3.2 Effect of BFs on Crops

#### 3.2.1 Plant Growth

Figure 2 shows the results from fertilization trials on lettuce, ryegrass, and radish subjected to various BFs treatments. Overall, fresh- and dry-weight measurements across the three crops indicated that all biofertilizers increased plant biomass relative to the unfertilized control. However, the degree of improvement varied depending on the specific treatment and crop examined.

**Figure 2:**
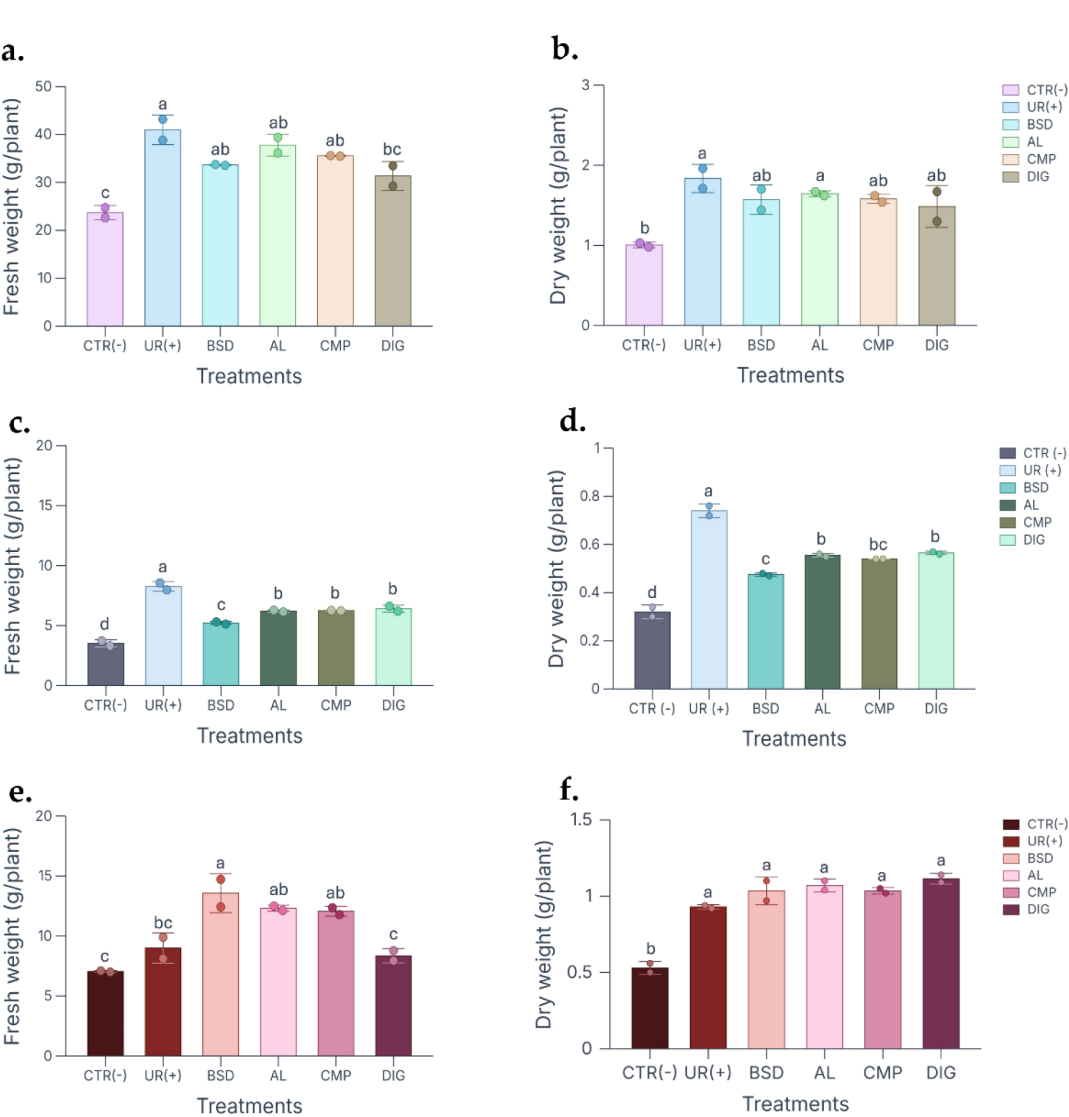
Effects of biofertilizer application on the growth of lettuce, ryegrass, and radish, measured by a) fresh weight and b) dry weight for lettuce; c) fresh weight and d) dry weight for ryegrass; and e) fresh weight and f) dry weight for radish. Control (CTR, unfertilized), urea (positive control), biosolids (BSD), microalgae (AL), compost (CMP), and digestate (DIG). Bars indicate mean ± standard deviation, n = 4. The letters indicate significant differences between treatments for each crop.

The fresh and dry weights of both lettuce and ryegrass showed a consistent pattern across all treatments studied (Figure 2a–d). All BFs, except digestate, increased fresh and dry weight for both crops compared to the unfertilized control. Specifically, biosolids applied to lettuce led to a 42% increase in fresh weight and a 56% increase in dry weight relative to the control (Figure 2a and b), while in ryegrass, they enhanced both fresh and dry weight by 49% and 48%, respectively (Figure 2c and d). Notably, among the BFs, microalgae and compost outperformed biosolids in lettuce cultivation, yielding increases in fresh weight of 60% and 50%, and increases in dry weight of 64% and 57%, respectively (Figure 2a and b). In ryegrass, digestate, compost, and microalgae resulted in increases in fresh weight of 82%, 78%, and 77%, and dry weight increases of 77%, 69%, and 73%, respectively (Figure 2c and d). On the other hand, in ryegrass, urea differed significantly from all other treatments (*P* > 0.05). This suggests that ryegrass, which are characterized by short growth cycles and high nutrient demands during early development, benefited from fertilizers that supply readily available nitrogen, such as urea, microalgae, digestate, and compost, compared to biosolids, which likely exhibit a more gradual nutrient release.^38,39^ The better performance observed with microalgae and compost compared to biosolids in lettuce may be attributed to the rapid mineralization of nutrients. In the case of microalgae, this may also result from the presence of various bioactive molecules and polysaccharides that promote rapid shoot growth.^6^ Conversely, compost may provide a balanced nutrient profile by optimizing the C/N ratio, thereby facilitating rapid nitrogen release during early crop growth stages.^30^ In contrast, biosolids release nutrients gradually due to their higher organic nitrogen content, which may restrict the growth of short-cycle crops.^40^

A different trend was observed in fresh weight of radish (Figure 2e). Several treatments increased fresh biomass compared with the unfertilized control, with significant differences observed for biosolids, microalgae, and compost. These treatments showed the best performance, increasing fresh weight by 93%, 75%, and 71%, respectively (*P* > 0.05). In contrast, digestate did not differ significantly from the controls. Conversely, when evaluating the dry weight of radishes, no significant differences were observed among the treatments (*P* > 0.05) (Figure 2f), although urea exhibited values similar to those of the BFs treatments. This may relate to the rapid release of mineral nitrogen, which accelerates biomass accumulation, whereas BFs, such as biosolids, release nitrogen gradually, maintaining growth without marked increases in dry matter.^41^ Similar results have been reported when comparing compost-fertilizers and biosolids with mineral fertilizers, with fresh biomass values showing more pronounced differences than dry mass.^42^

From a practical agronomic perspective, crop productivity remains the primary driver of fertilization decisions, often taking precedence over environmental considerations or potential biostimulant effects. In this context, crop responses suggest that STW-derived biosolids may be most effective when integrated with readily available nutrient sources, particularly in short-cycle systems with high early nitrogen demand. While mineral fertilizers can promote rapid biomass accumulation, biosolids provide a more gradual nutrient supply, which may improve nitrogen retention and better synchronize with plant uptake over time. This complementary approach supports the maintenance of yield targets while simultaneously addressing nutrient efficiency and reducing losses, highlighting the potential role of biosolids within integrated nutrient management strategies rather than as direct one-to-one replacements for conventional fertilizers.^35,40^

#### 3.2.2 Protein Content

Figure 3 presents the protein content of the crops studied, providing supporting evidence for the results obtained in the fertilization trials. Beyond its role in determining the nutritional value of vegetable products, protein content is a reliable indicator of nitrogen uptake efficiency and crop physiological status. For lettuce (Figure 3a), the urea treatment showed the highest protein levels, whereas the microalgae treatment reached levels comparable to those of urea. Overall, all treatments exceeded the negative control, indicating that added nitrogen, even from slow-release sources, can increase protein accumulation in crops such as lettuce. A similar pattern emerged for ryegrass (Figure 3b), with urea treatment exhibiting significantly higher protein content than the other treatments. The remaining biofertilizers, including biosolids and microalgae, showed values comparable to the negative control. This suggests that although organo-mineral nitrogen forms in biosolids may support protein synthesis, their slower release does not produce the immediate increase observed with readily available mineral nitrogen. Consequently, while biosolids may not maximize biomass production under short-cycle conditions, they can sustain adequate nitrogen availability for protein formation.^43^ In contrast, compost promoted greater vegetative biomass development relative to biosolids, likely due to its gradual nitrogen release and beneficial effects on soil structure.^11^ However, this may have caused a dilution effect,^44^ resulting in a lower protein concentration than in biosolids. Digestate showed a similar behavior, likely reflecting a lower capacity to enhance protein synthesis.

**Figure 3:**
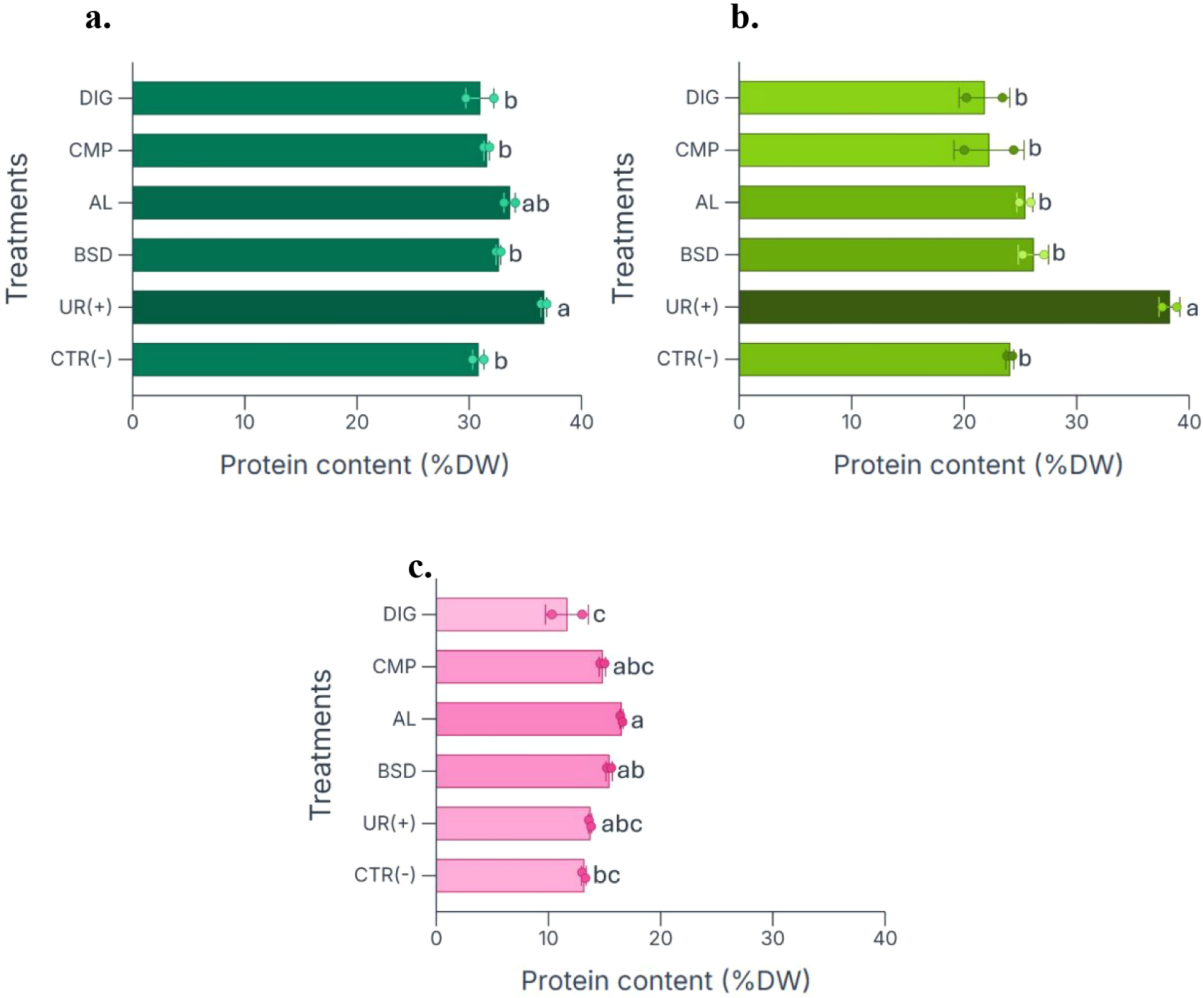
Protein content in crops of a) lettuce, b) ryegrass, and c) radish. Control (CTR, unfertilized control), urea (positive control), biosolids (BSD), microalgae (AL), compost (CMP), and digestate (DIG). DW: dry weight. The bars represent the mean ± standard deviation, n = 4. Different letters indicate significant differences between treatments.

The protein content in the radish trial showed a different pattern (Figure 3c). In this case, the microalgae treatment yielded the highest protein content, followed by biosolids, while compost and urea showed intermediate values. Digestate presented the lowest protein concentration. Compared with the unfertilized control, significant differences were primarily attributable to the microalgae treatment. The positive response to microalgae may be attributable to their high amino acid content and their stimulation of plant nitrogen metabolism.^6^ Biosolids also exhibited competitive protein levels, possibly due to a balanced nutrient profile that combines readily available and slowly mineralizing nitrogen forms.^45^

Overall, biosolids and microalgae showed consistent potential as biofertilizers, although their effectiveness varied by crop type. While mineral nitrogen produced the most pronounced response in ryegrass, biosolids performed comparably in radish and maintained adequate protein levels across crops. These findings suggest that crop-specific fertilization strategies could reduce reliance on inorganic nitrogen inputs, positioning STW-derived biosolids as a viable component of sustainable nutrient management systems.

### 3.3 Nitrogen Leaching Tests

Soluble N levels (ammonium-N, nitrite-N, and nitrate-N) were analyzed through a leachate monitoring test to evaluate the potential for groundwater contamination following BFs application to the soil (Figures 4, 5, and 6). The leaching results showed significant differences among treatments and crop types.

**Figure 4:**
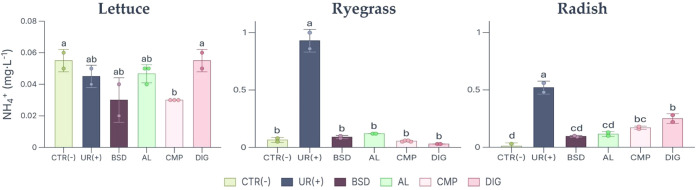
Concentrations of ammonium (NH_4_^+^) in the leachate from each treatment: control (CTR, unfertilized control), urea (positive control), biosolids (BSD), microalgae (AL), compost (CMP), and digestate (DIG). The bars show the mean ± standard deviation, n = 4. Different letters indicate significant differences among the treatments.

**Figure 5:**
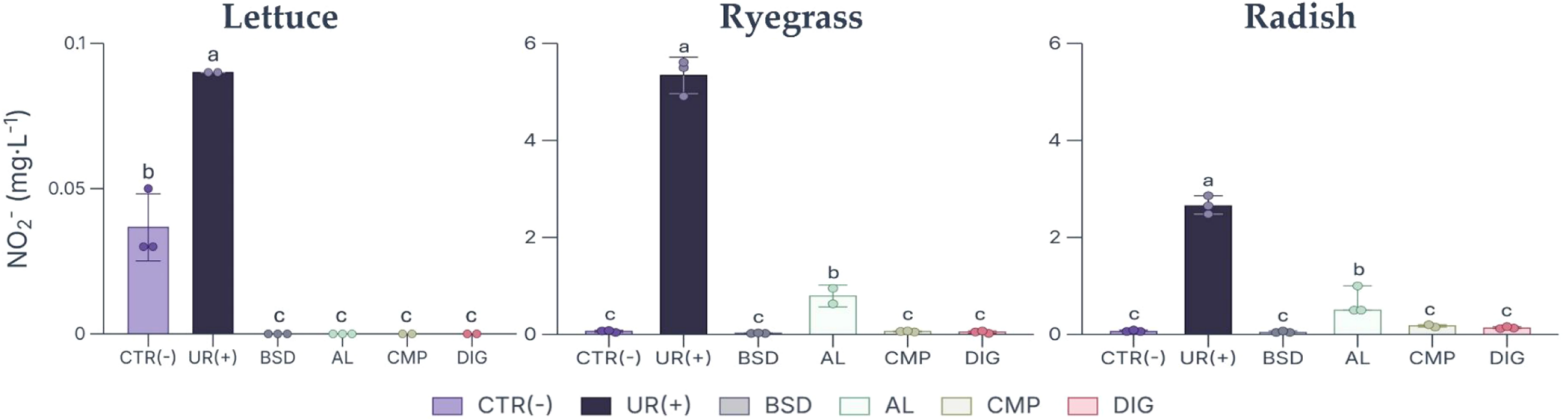
Concentrations of nitrite (NO_2_^–^) in the leachate from each treatment: control (CTR, unfertilized control), urea (positive control), biosolids (BSD), microalgae (AL), compost (CMP), and digestate (DIG). The bars show the mean ± standard deviation, n = 4. Different letters indicate significant differences among the treatments.

**Figure 6:**
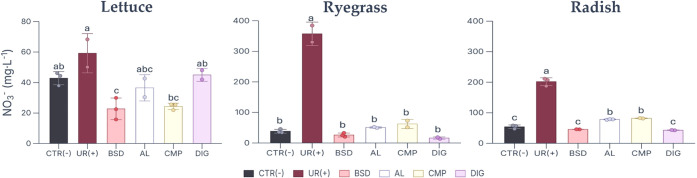
Concentrations of nitrate (NO_3_^–^) in the leachate from each treatment: control (CTR, unfertilized control), urea (positive control), biosolids (BSD), microalgae (AL), compost (CMP), and digestate (DIG). The bars show the mean ± standard deviation, n = 4. Different letters indicate significant differences among the treatments.

For ammonium-N (NH_4_^+^), the highest losses were observed in the urea treatments, particularly in ryegrass (0.93 mg·L^–1^ NH_4_^+^), followed by radish (0.52 mg·L^–1^ NH_4_^+^) and lettuce (0.04 mg·L^–1^ NH_4_^+^). In lettuce crops, biosolids and compost had the lowest NH_4_^+^ leaching (0.03 mg·L^–1^ NH_4_^+^). In comparison, AL and DIG showed higher values (0.05 mg·L^–1^ NH_4_^+^ and 0.06 mg·L^–1^ NH_4_^+^, respectively) (Figure 4). In ryegrass, DIG had the lowest NH_4_^+^ leaching (0.03 mg·L^–1^ NH_4_^+^). In contrast, BSD and AL showed moderate NH4+ concentrations (0.05 mg·L^–1^ and 0.06 mg·L^–1^, respectively). In radish, urea again produced the highest NH_4_^+^ leachate (0.03 mg·L^–1^ NH_4_^+^), while BSD was among the lowest. This pattern indicates that BSD, compared to other BFs treatments, releases nitrogen gradually, thereby reducing NH_4_^+^ losses and synchronizing availability with plant uptake.^45^

Regarding nitrite (NO_2_^–^), leaching was generally lower with respect to the other N mineral forms studied. However, the urea treatment still showed the highest leaching levels (Figure 5). In lettuce, only urea exhibited detectable leaching (0.09 mg·L^–1^ NO_2_^–^). For ryegrass, nitrite losses from urea and AL were observed at 5.34 mg·L^–1^ NO_2_^–^ and 0.79 mg·L^–1^ NO_2_^–^, respectively, while BSD and CMP showed negligible values. In radish, urea again led in nitrite loss, followed by AL, CMP, and DIG. The absence of NO_2_^–^ losses in crops treated with BSD may indicate a more stable nitrification process, in which nitrogen is transformed and immobilized by soil microorganisms rather than lost from the soil. This observation aligns with previous studies indicating that stable organic matter can regulate nitrite accumulation by moderating nitrifying activity.^39,45^

Significant differences were observed in nitrate (NO_3_^–^) levels in the leachates (Figure 6). In lettuce, urea resulted in the highest NO_3_^–^ losses (56.22 mg·L^–1^ NO_3_^–^). In contrast, BSD outperformed all other treatments, resulting in lower levels than the other BFs. In ryegrass, urea caused the most significant leaching loss (357.29 mg·L^–1^ NO_3_^–^), while BSD and DIG showed the lowest values (26.12 mg·L^–1^ NO_3_^–^ and 16.14 mg·L^–1^ NO_3_^–^, respectively). In radish, urea treatment showed a value of 201.95 mg·L^–1^ NO_3_^–^, followed by AL and CMP, with DIG and BSD presenting the lowest levels. This pattern highlights the stability of BSD derived from STW, thereby reducing the risk of NO_3_^–^ leaching, which is a crucial environmental benefit given the connection between agricultural nitrate losses and groundwater contamination.^46^

Overall, integrating crop fertilization trials results with leaching data emphasizes the dual role of BSD as BFs that promote plant growth, including protein content, while decreasing nitrogen losses. Unlike urea, which maximizes yield but may cause high nutrient leaching, STW-derived BSD provides a slower, more controlled nutrient release, lowering environmental risk and aiding sustainable soil fertility management.

### 3.4 Biostimulant Effects of Substrate Extracts

#### 3.4.1 Germination Index of Cress Seeds

Figure 7a shows the germination index (GI) of cress seeds evaluated at two different concentrations of substrate extracts (20 mg·L^–1^ DOC and 200 mg·L^–1^ DOC), compared with a negative control (deionized water) (CTR (−)). According to the literature, a GI value above 80% indicates no phytotoxicity, while a value over 120% suggests a biostimulant effect.^29^ Based on this criterion, neither concentration (20 mg·L^–1^ DOC or 200 mg·L^–^1 DOC) of BSD showed a toxic effect on cress, as the GI remained above the 80% threshold. Specifically, the BSD extract increased the GI at both concentrations compared to the control, with increases of 144.8% at 20 mg·L^–1^ and 149.6% at 200 mg·L^–1^, demonstrating a biostimulant effect. These findings align with those of Cano-Larrotta et al.^10^ which indicated that biosolids derived from STW do not exhibit phytotoxic effects on lettuce seeds.

**Figure 7:**
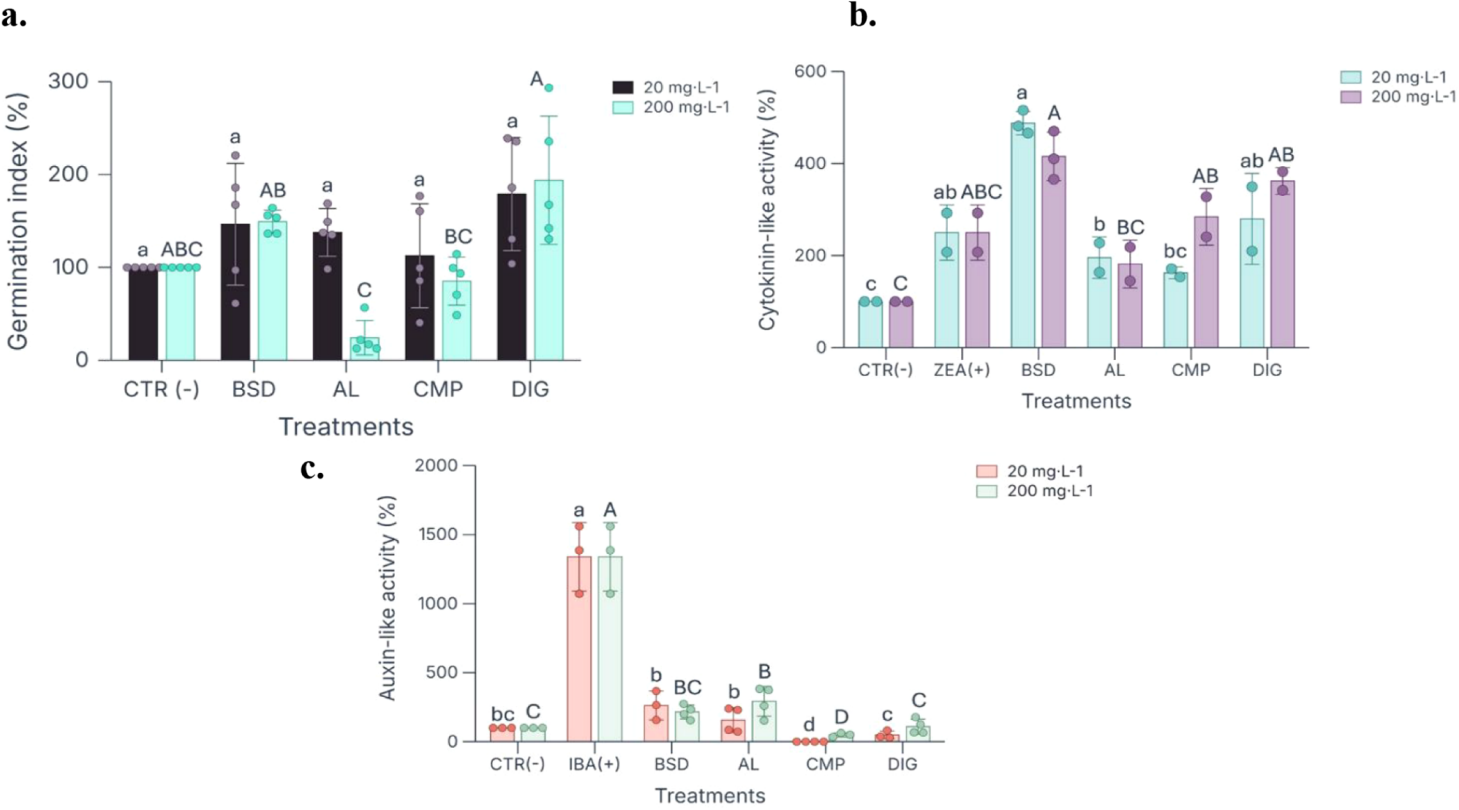
Biostimulant assays using biofertilizer extracts from negative control (CTR (−), deionized water), biosolids (BSD), microalgae (AL), compost (CMP), and digestate (DIG) at two concentrations: 20 mg·L^–1^ DOC and 200 mg·L^–1^ DOC. (a) Germination index in cress seeds; (b) inhibition of chlorophyll degradation in wheat leaves compared to a positive control using the cytokinin zeatin at 1 ppm; (c) secondary root formation in bean seedlings compared to a positive control using indole-3-butyric acid at 1 ppm. Deionized water was used as a negative control in all assays. Each bar represents the mean ± standard deviation (n = 5). Different letters indicate significant differences. Lowercase letters indicate the ANOVA results for the 20 mg·L^–1^ DOC concentration, while capital letters indicate the 200 mg·L^–1^ DOC concentration results.

For the other substrates, no treatments showed toxicity at the 20 mg·L^–1^ concentration. In fact, at this concentration, DIG extracts exhibited the highest GI value (177.3%), likely due to the presence of hormone-like substances in this type of organic material, similarly to compost.^47^ Although compost did not show phytotoxic effects, it did not appear to exert a biostimulant effect (Figure 7a). Microalgae did not display phytotoxicity at 20 mg·L^–1^. However, at 200 mg·L^–1^, the microalgae extracts demonstrated a phytotoxic effect, with a GI value of 22.4%, compared to the other tested extracts. This could be due to the degradation of biostimulant substances within the microalgae^48^ or to the high phenolic compounds content, which might inhibit germination at higher doses, as observed in literature.^11^

#### 3.4.2 Chlorophyll Retention Assay on Wheat Leaves

The function of cytokinin hormones was examined by applying substrate extracts to wheat leaf tissues and observing chlorophyll retention capacity. This study aimed to demonstrate that extracts, particularly the biosolids extract, can delay chlorophyll breakdown by containing cytokinin-like compounds and prevent senescence. The results of this assay are shown in Figure 7b. The wheat leaf senescence assay revealed that BSD extracts were more effective at delaying chlorophyll loss than the commercial cytokinin zeatin, at both 20 mg·L^–1^ and 200 mg·L^–1^. In contrast, AL, CMP, and DIG extracts showed slightly lower activity compared to the zeatin reference.

Biological activity observed following biosolids application may be associated with bioactive molecules previously reported in treated sewage sludge and other organic biomass, including natural cytokinins, peptides, phenols, and humic-like compounds.^49^ Although some of these molecules were not directly analyzed in the present study, several studies have shown that humic-like fractions can exhibit cytokinin-like activity and contribute to hormone stabilization and delayed senescence in plants.^50^ Similarly, applications of treated sewage sludge have been linked to increased cytokinin levels and delayed senescence in grass.^51^ Likewise, organic amendments derived from compost have been reported to contain detectable cytokinins, albeit at lower concentrations and with more variable effects.^52^ Recent sludge valorization studies have further provided quantitative evidence of phytohormone pools in sludge-derived matrices; for instance, thermal hydrolysis-anaerobic digestion has been reported to yield approximately 80 mg·kg^–1^ VS of phytohormones in digested sludge, and alkaline hydrothermal treatment has produced liquid fractions in which individual phytohormones can reach ∼10^4^ μg·L^–1^ under optimized conditions.^53,54^ Collectively, these sludge-based observations support an associative interpretation of the biostimulant responses observed here and motivate future work to profile and quantify specific cytokinin forms and humic-associated bioactive compounds in STW-derived biosolids.

#### 3.4.3 Adventitious Root Induction in Mung Beans

Among the phytohormones, auxins are paramount, as they regulate root initiation, elongation, and overall root development.^55^ The auxin assay conducted in this study facilitates the evaluation of the auxin-like activity of substrate extracts compared to a synthetic auxin, i.e., indole-3-butyric acid (IBA), which served as the positive control, alongside a negative control using distilled water. Figure 7c presents the results regarding the capacity to induce root formation, measured as root biomass at two evaluated concentrations (20 mg·L^–1^ DOC and 200 mg·L^–1^ DOC).

As expected, the IBA control showed the strongest promotion of root formation at both concentrations. Among the substrate extracts, BSD exhibited the most consistent auxin-like activity. At a concentration of 20 mg·L^–1^ DOC, BSD and AL significantly promoted root development compared to the other substrates. In contrast, CMP and DIG demonstrated very low activity, with CMP showing almost no evidence of auxin-like activity. At 200 mg·L^–1^ DOC, AL slightly outperformed BSD. However, these two treatments remained the most effective among the analyzed substrate extracts, while CMP and DIG yielded only minor increases compared to the control. The pronounced activity of BSD suggests they could function similarly to AL extracts. Indeed, according to this study, BSD showed better results than AL compared to recent literature,^29,56^ which are recognized as potent biostimulants in agriculture.^6^ Biosolids are known to contain indolic precursors and humic fractions capable of activating auxin signaling and stimulating root development.^57^ The consistent results of BSD across both concentrations emphasize the potential of STW to produce stable, active biosolids with effects comparable to those of AL, while offering a sustainable and cost-effective alternative to commercial products. Conversely, the slow response observed with CMP and DIG may be attributed to their limited content of bioactive compounds.^52,58^

### 3.5 Regulatory and Policy Considerations for STWs’ Biosolids Reuse

Beyond agronomic performance, these findings also have implications for waste management and agricultural policy frameworks. From a policy perspective, the valorization of biosolids from STWs aligns with European strategies to promote the circular economy and sustainable nutrient management.^59^

Regulation (EU) 2019/1009 establishes a framework for the use of treated organic residues as fertilizing products, provided they meet strict requirements for stabilization, safety, and contaminant thresholds, including limits for heavy metals and pathogens. Although sewage sludge is not explicitly authorized as a fertilizing product under this regulation, the emphasis on nutrient recovery from waste streams and the use of secondary raw materials opens a potential regulatory pathway for appropriately treated sludge-derived materials. In parallel, the EU Circular Economy Action Plan highlights nutrient recycling from wastewater and organic wastes as a key strategy to reduce dependency on mineral fertilizers and improve resource efficiency.^59^

In this context, STW biosolids, characterized by biological stabilization processes and low levels of pathogens and heavy metals,^10^ could be considered within existing policy frameworks as part of integrated waste management and agricultural strategies. Previous studies have highlighted the role of treated biosolids in closing nutrient loops while maintaining acceptable environmental risk profiles when quality standards are respected.^60–62^

The incorporation of STW biosolids alongside conventional fertilization practices could help reduce reliance on mineral fertilizers, improve nutrient use efficiency, and support waste-to-resource approaches, particularly when applied under controlled conditions and in compliance with regulatory quality criteria. Such integration would align with broader EU objectives for sustainable agriculture and nutrient circularity, while offering decentralized wastewater treatment systems an additional valorization route for residual biomass.

## 4 Conclusions

This study proved that biosolids-derived STW may offer a sustainable alternative to synthetic fertilizers for selected crops. Across the three crops examined, biosolids-derived STW increased yield and protein content relative to unfertilized controls and outperformed other commonly used biofertilizers. Their application consistently resulted in lower nutrient leaching than urea, highlighting their potential to reduce environmental risks while maintaining soil fertility. Beyond their fertilizing capabilities, biosolids-derived STW showed significant bioactive potential. These findings suggest biosolids possess both biofertilizing and biostimulant properties, offering a low-cost strategy to reduce reliance on energy-intensive synthetic fertilizers and expensive plant growth regulators. Future studies should focus on testing higher concentrations of these biosolids to better quantify hormonal activity, fractionate the extracts to identify the specific compounds responsible for bioactivity, and validate these results through field-scale experiments across diverse soil and climate conditions. Additionally, scaling up agronomic trials will be crucial for assessing the effects of biosolids on soil health.

## Supplementary Material



## Data Availability

Data will be made available upon request.

## Notes

Physicochemical properties of soil used in the biofertilizers assay: methods and results (Table S1) (DOCX)
