# Supplementary material for: From Waste to Resource: Biosolids from Sludge Treatment Wetlands as Biofertilizers and Biostimulants
Source: ACS Environ Au. 2026 Apr 21;6(4):702–14. doi: 10.1021/acsenvironau.6c00070 (PMC13377512; doi:10.1021/acsenvironau.6c00070)
Supplement: Supplementary file 1 [file vg6c00070_si_001.docx]

Supporting information for:

**From waste to resource: biosolids from sludge treatment wetlands as biofertilisers and biostimulants**

Ana Cano-Larrotta^a^, Luisa Massaccesi^b^, Enrica Uggetti^a*^ and Mirko Cucina^b^

^a^GEMMA-Group of Environmental Engineering and Microbiology, Department of Civil and Environmental Engineering, Universitat Politècnica de Catalunya-BarcelonaTech, c/Jordi Girona 1-3, Building D1, E-08034 Barcelona, Spain

^b^National Research Council of Italy, Institute for Agricultural and Forest Systems in the Mediterranean, Via della Madonna Alta 128, 06123, Perugia, Italy

*Email: enrica.uggetti@upc.edu

**Table S1.** Physicochemical properties of soil used in the biofertilisers assay: methods and results. Data are reported on dry mass weight.

| **Parameters** | **Units** | **Soil** |
| --- | --- | --- |
| Gross Sand | % | 6.4 |
| Fine Sand | % | 26 |
| Silt | % | 37.9 |
| Clay | % | 29.7 |
| Texture | - | Clayey-Loam |
| pH |  | 7.8 |
| EC | mS/cm | 0.23 |
| Total organic C | % | 1.2 |
| Total N | % | 0.19 |
| Total S | % | 0.04 |
| Available P | mg kg^-1^ | 26 |
| Exchangeable K | mg kg^-1^ | 219 |
| Humidity | % | 11.9 |
| Water holding capacity | % | 36.4 |
|  |  |  |
| **Analytical procedures for soil analysis:**  Soil texture analysis was determined according to the classical Andreasen method. The pH and electrical conductivity (EC) of the soil were measured using an electrode in a soil-water ratio of 1:5 (w/v) (pH-Meter Basic 20+, Crison Instruments, Barcelona, Spain; conductivity probe Ec-Meter Basic 30+, Crison Instruments, Barcelona, Spain). Soil organic carbon (C), soil nitrogen (N), and the C/N ratio were analyzed using an elemental analyzer (MacroCUBE CNHS, Elementar Italia, Lomazzo, Italy) after removing carbonates with HCl (10% v/v). Water holding capacity was determined using the sandbox method. Available P was determined by the Olsen method. Exchangeable K was determined by soil extraction with ammonium acetate and following determination of K in the extract by inductively coupled plasma mass spectrometry (Varian, Fort Collins, CO, USA). | | |
